# Change in full immunization inequalities in Indian children 12–23 months: an analysis of household survey data

**DOI:** 10.1186/s12889-021-10849-y

**Published:** 2021-05-01

**Authors:** Brian Wahl, Madhu Gupta, Daniel J. Erchick, Bryan N. Patenaude, Taylor A. Holroyd, Molly Sauer, Madeleine Blunt, Mathuram Santosham, Rupali Jayant Limaye

**Affiliations:** 1 Department of International Health, Johns Hopkins Bloomberg School of Public Health, 415 N Washington Street, Floor 5, Baltimore, MD 21231 USA; 2 International Vaccine Access Center, Johns Hopkins Bloomberg School of Public Health, Baltimore, USA; 3 Department of Community Medicine and School of Public Health, Postgraduate Institute of Medical Education and Research, Chandigarh, India; 4 Department of Epidemiology, Johns Hopkins Bloomberg School of Public Health, Baltimore, USA; 5 Department of Health, Behavior & Society, Johns Hopkins Bloomberg School of Public Health, Baltimore, USA

**Keywords:** Immunization, Inequalities, India, Child health

## Abstract

**Background:**

India has made substantial progress in improving child health in recent years. However, the country continues to account for a large number of vaccine preventable child deaths. We estimated wealth-related full immunization inequalities in India. We also calculated the degree to which predisposing, reinforcing, and enabling factors contribute to these inequalities.

**Methods:**

We used data from the two rounds of a large nationally representative survey done in all states in India in 2005–06 (*n* = 9582) and 2015–16 (*n* = 49,284). Full immunization status was defined as three doses of diphtheria-tetanus-pertussis vaccine, three doses of polio vaccine, one dose of Bacillus Calmette–Guérin vaccine, and one dose of measles vaccine in children 12–23 months. We compared full immunization coverage by wealth quintiles using descriptive statistics. We calculated concentration indices for full immunization coverage at the national and state levels. Using predisposing, reinforcing, and enabling factors associated with full immunization status identified from the literature, we applied a generalized linear model (GLM) framework with a binomial distribution and an identity link to decompose the concentration index.

**Results:**

National full immunization coverage increased from 43.65% in 2005–06 to 62.46% in 2015–16. Overall, full immunization coverage in both 2005–06 and 2015–16 in all states was lowest in children from poorer households and improved with increasing socioeconomic status. The national concentration index decreased from 0.36 to 0.13 between the two study periods, indicating a reduction in poor-rich inequality. Similar reductions were observed for most states, except in states where inequalities were already minimal (i.e., Tamil Nadu) and in some northeastern states (i.e., Meghalaya and Manipur). In 2005–06, the contributors to wealth-related full immunization inequality were antenatal care, maternal education, and socioeconomic status. The same factors contributed to full immunization inequality in 2015–16 in addition to difficulty reaching a health facility.

**Conclusions:**

Immunization coverage and wealth-related equality have improved nationally and in most states over the last decade in India. Targeted, context-specific interventions could help address overall wealth-related full immunization inequalities. Intensified government efforts could help in this regard, particularly in high-focus states where child mortality remains high.

## Background

India has made substantial progress in improving child health in the last two decades. However, the country continues to account for a disproportionate burden of the global morbidity and mortality in children less than five years. Despite having 17% of the global under-five population, 27% of deaths in this age group in 2018 were in India [1, 2]. A substantial proportion of mortality in children in India is vaccine preventable [3].

Only 63% of children in India were fully immunized—i.e., three doses of diphtheria-tetanus-pertussis (DTP) vaccine, three doses of polio vaccine, one dose of Bacillus Calmette–Guérin (BCG) vaccine, and one dose of measles vaccine—according to the National Family Health Survey (NFHS) conducted in 2015–16 [4]. National averages mask subnational disparities in vaccine coverage. States in the south consistently report higher full vaccine coverage compared to those in the north [4]. Several studies have described differences in immunization coverage by socioeconomic status, access to antenatal care, caste, religion, gender, and urban/rural settings [5–8].

The national immunization program aims to provide free-of-cost vaccines to an annual birth cohort of approximately 27 million children—more than any other country [2]. India is also one of the largest countries by area with significant variations in geographies and population densities making the delivery of vaccines especially challenging. Compounding these delivery challenges, poor program monitoring and weak disease surveillance infrastructure in some areas make it difficult to identify and address areas for immunization program strengthening [9]. These factors have contributed to an inability to reach vulnerable populations, particularly those in poor rural areas [10, 11].

The Government of India launched the National Rural Health Mission (NRHM) in 2005, now the National Health Mission (NHM), to expand and improve access to many primary health services for women and children in the country [12, 13]. The government also recently initiated programs specifically targeted at increasing immunization coverage. Mission Indradhanush was launched in 2014 with the goal of achieving full immunization coverage above 90% over several phases throughout the entire country [14]. However, this target has not yet been met and an intensified version of Mission Indradhanush continues to work toward improving immunization coverage in India—see panel.

As full immunization coverage increases, it is increasingly imperative to understand the status of immunization inequalities. The PRECEDE-PROCEED model is a useful lens through which to assess immunization status toward informing program planning efforts. The model focuses on predisposing, reinforcing, and enabling factors that contribute to health poor status (i.e., not being immunized) [15]. This model can help to identify behavioral interventions that can address alterable causes of immunization inequality. Where immutable contributors are identified, programs can be oriented to prioritize at-risk communities and individuals. Using this framework, we assessed the change in wealth-related inequalities in full immunization coverage at the national, regional, and state level in India from 2005–06 to 2015–16 and quantified the degree to which various factors contribute to these inequalities.

**Table:** 

**Panel: National programs** **National Health Mission:** The National Health Mission (NHM), India’s flagship health program, was formed in 2013 by merging two other missions—the National Rural Health Mission (NHRM) established in 2005 and National Urban Health Mission (NUHM). The goal of NHM is to achieve universal access to equitable, affordable, and quality health care services accountable and responsive to the needs of all Indians. NHM has organized its efforts in several broad areas that include reproductive, maternal, neonatal, child and adolescent health; health systems; non-communicable diseases; communicable diseases; and infrastructure maintenance. Although led by the central government, NHM operates by providing financial and technical support to state governments, which are responsible for the delivery of health services. Approximately half of India’s health budget was allocated to NHM in 2019, with the remainder provided to other major government programs for health insurance and research. **Universal Immunization Programme:** Originally established by the Ministry of Health and Family Welfare in 1978 as the Expanded Programme on Immunization (EPI), and renamed in 1985, the Universal Immunization Programme (UIP) provides routine immunization services to infants, children, and pregnant women in India. The UIP currently includes the following vaccines in all states and UTs: Bacille Calmette-Guérin (BCG), diphtheria- tetanus-pertussis (DTP) booster, fractional dose inactivated polio vaccine (fIPV), hepatitis B vaccine, measles-rubella (MR) vaccine, oral polio vaccine (OPV), pentavalent vaccine (DTP, hepatitis B, and *Haemophilus influenzae* type b [Hib]), rotavirus vaccine (RVV), and tetanus toxoid (TT). Additionally, the UIP includes two vaccines that are not presently used in all states/union territories: pneumococcal conjugate vaccine (PCV) has been introduced in a subset of states and plans are in place to scale up nationally; and Japanese encephalitis (JE) vaccine is used in endemic districts. **Mission Indradhanush:** In 2014, Mission Indradhanush was launched by the Ministry of Health and Family Welfare (MOHFW) to increase full immunization coverage for all children to 90% by 2020. During four phases, Mission Indradhanush targeted 528 districts with low coverage and underserved, hard-to-reach populations. The program employed multiple strategies, including strengthening microplanning, monitoring, social mobilization, and vaccine delivery systems. MOHFW credits the first phase of Mission Indradhanush—which operated from April 2015 to July 2017—with increasing full immunization by an absolute 7%. In 2017, India announced the Intensified Mission Indradhanush—an expanded effort to achieve 90% full immunization coverage by 2018. Intensified Mission Indradhanush retained the same strategies with the addition of increasing the focus on urban areas, involving non-health sectors in social mobilization activities, and providing financial support. In late 2019, this program was relaunched and continues as Intensified Mission Indradhanush 2.0.	

## Methods

### Data sources

We used data from the two most recent rounds of the National Family Health Survey (NFHS). The study period was 2005–06 (NFHS-3) [16] and 2015–16 (NFHS-4) [4]. NHFS is a nationally representative cross-sectional household survey administered by the Ministry of Health and Family Welfare (MOHFW) in India with coordination and technical guidance provided by the International Institute for Population Science (IIPS) based in Mumbai. NFHS-3 and NFHS-4 collected data from a population representative sample of 109,041 households in 29 states and union territories and 601,509 households in 36 states and union territories, respectively. NFHS-4 provides data at the district and state levels, while NFHS-3 only includes data at the state level. NFHS-3 and NFHS-4 samples are both two-stage stratified samples. Census data were used as the sampling frame for primary sampling units (i.e., villages in rural areas and census enumeration blocks in urban areas).

### Variables

We assessed full immunization status in children 12–23 months. Children were considered fully immunized if they received three doses of diphtheria-tetanus-pertussis (DTP3) vaccine, three doses of polio vaccine, one dose of Bacillus Calmette–Guérin (BCG) vaccine, and one dose of measles vaccine. Children were considered not fully vaccinated if they had missed any or all of these vaccinations. Immunization status in both NFHS surveys was based on immunization cards or by parental recall if cards were not available.

**Fig. 1:**
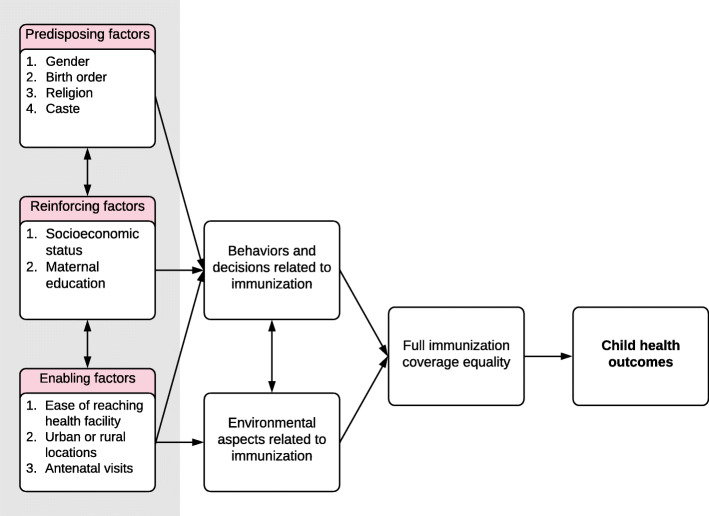
PRECEDE/PROCEED framework for full immunization coverage, including predisposing, reinforcing, and enabling factors in India as informed by the literature

To help inform intervention planning models, we used the PRECEDE/PROCEED framework to identify factors contributing to immunization coverage [15, 17]. Specifically, we identified predisposing, reinforcing, and enabling factors associated with full immunization status from the literature. These factors relate to each other and contribute to broader behaviors and environmental aspects related to immunization (Fig. 1).

Predisposing factors included gender, birth order, religion, and caste. Birth order was considered ordinal. We grouped children with a birth order of five or more together, as the proportion of those with any birth order greater than five was less than 5%. We created three categories to define religion: Hindu, Muslim, or other. NFHS survey use four broad classifications for caste. These included scheduled caste and scheduled tribe (SC/ST), other backward caste (i.e., a collective term used by the Government of India to characterize castes that are considered socially disadvantaged), and other caste. Other caste is a heterogeneous group traditionally viewed as having higher social status.

Reinforcing characteristics included socioeconomic status and maternal education. We define socioeconomic status using national wealth quintiles from each survey. NFHS-4 included state-level wealth quintiles. We used principal component analysis to develop state-level wealth quintiles for NFHS-3 using a method described elsewhere [18]. The highest level of education reported by the mother was used to define educational attainment in both survey rounds. We retained the original survey response categories: no education, incomplete primary education, complete primary education, incomplete secondary education, complete secondary education, and higher education.

Enabling factors included ease of reaching a health facility, geographic location, and the number of antenatal visits. Ease of accessing a health center was defined by respondent perceptions and included “no problem,” “not a big problem,” and “big problem” in accessing a health facility. Geographic location was defined as either urban or rural. We excluded *non de jure* residents. Antenatal visits were categorized as those with no antenatal visits, those with insufficient visits (i.e., less than four), and those with sufficient visits (i.e., four or more).

### Data analysis

To describe inequalities by socioeconomic status, full immunization coverage was disaggregated by wealth quintile at the national and state levels. Except for Delhi, we do not report results individually for union territories given their small populations. Telangana separated from Andhra Pradesh in June 2014 and therefore immunization coverage for Telangana and Andhra Pradesh is reported together for 2005–06 but separately for 2015–16. We calculated absolute differences and relative measures of full immunization coverage using the lowest (i.e., poorest) and highest (i.e., richest) wealth quintiles to describe coverage inequalities in 2005–06 and 2015–16 in each state.

We used the concentration index to measure inequalities in full immunization coverage for 2005–06 and 2015–16. The method is described elsewhere [19–22]. In brief, the concentration index is a summary measure of inequality where, in this case, a positive value indicates full immunization is distributed in favor of those in the higher wealth quintiles. A negative concentration index would suggest that full immunization is distributed in favor of those in the lower wealth quintiles. A larger concentration index corresponds with greater inequality in either direction (i.e., favoring the rich or the poor). The concentration index is bound by −1 and 1. A concentration index of 0 represents equality. We used Errerygers correction when calculating the concentration index [20]. The delta method was used to obtain the standard error [23].

We used a regression decomposition approach to determine the contribution of each factor to the concentration index for full immunization. Contributing factors include predisposing, reinforcing, and enabling determinants for full immunization. We also categorized states and union territories into one of six geographically contiguous and socioeconomically similar regions described in Table 1.

**Table 1:** Indian states by region and categories

Region	States
Central	Chhattisgarh, Madhya Pradesh, Rajasthan, Uttar Pradesh
East	Bihar, Jharkhand, Odisha, West Bengal
North	Chandigarh, Delhi, Haryana, Himachal Pradesh, Jammu & Kashmir, Punjab, Uttarakhand
Northeast^a^	Arunachal Pradesh, Assam, Manipur, Meghalaya, Mizoram, Nagaland, Sikkim, Tripura
South	Andaman & Nicobar Islands, Andhra Pradesh, Karnataka, Kerala, Lakshadweep, Puducherry, Tamil Nadu
West	Dadra & Nagar Haveli, Daman & Diu, Goa, Gujarat, Maharashtra
Union territories	Andaman & Nicobar Islands, Chandigarh, Dadra & Nagar Haveli, Daman & Diu, Delhi, Lakshadweep, Puducherry
High-focus states^b^	Assam, Bihar, Chhattisgarh, Jharkhand, Madhya Pradesh, Odisha, Rajasthan, Uttar Pradesh, Uttarakhand

^a^Northeast states exclude Assam for estimates reported at the state-level; ^b^States with high infant mortality are designated as high-focus states by the Government of India

Since full immunization is binary, we adopted a generalized linear model (GLM) framework using a binomial distribution with an identity link for the decomposition, following a previously described method to ensure consistent estimates that do not vary by choice of reference group for dichotomous and categorical predictors [24]. Elasticities are generated from the coefficients of the GLM model and used to determine the contribution of each predictor to the overall concentration index. The concentration index can be decomposed following a previously described approach [25]. The decomposition of concentration index is presented below:

$$ CI={\sum}_k\left(\frac{\beta_k{\overline{x}}_k}{\mu }{CI}_k\right)+\frac{GCI_{\varepsilon }}{\mu } $$

Where *CI* is the overall concentration index, $\frac{\beta_k{\overline{x}}_k}{\mu }$ is the elasticity at the mean of determinant *k* with respect to mean full immunization *μ*, *CI*_*k*_ is the concentration index of determinant *k*, and *GCI*_*ε*_ is the generalized concentration index for the error term. All analyses were done using Stata version 15.1 (Stata Corp, TX).

## Results

Immunization status was assessed among 9582 children 12–23 months in 2005–06 and among 49,284 children in the same age group in 2015–16. Full immunization coverage estimates for India and 30 states in 2005–06 and 2015–16 are provided in Table 2. National full immunization coverage increased by a relative 43.09% (95% confidence interval [CI]: 36.71, 49.91%) from 43.65% (42.11, 45.19%) in 2005–06 to 62.46% (61.78, 63.13%) in 2015–16. In 2005–06, state immunization coverage ranged from as low as 20.95% (95% CI: 15.94, 25.97%) in Nagaland, a state in the Northeast, to 80.89% (75.24, 86.54%) in Tamil Nadu, a state in the South. Nagaland had the lowest full immunization coverage in 2015–16 with 36.59% (95% CI: 32.54, 40.64) and Punjab in the North had the highest full immunization coverage with 89.05% (86.62, 91.47).

**Table 2:** National and state full immunization coverage inequalities for 2005–06 and 2015–16

State	Year	% Coverage (95% CI)	Wealth quintile 1 coverage (95% CI)	Wealth quintile 5 coverage (95% CI)	Difference^**a**^ (95% CI)	Ratio^**b**^ (95% CI)
Andhra Pradesh	2005–06	45.98% (39.37–52.58)	36.74% (23.06–50.42)	69.19% (53.07–85.30)	32.44 (11.10–53.79)	1.88 (1.21–2.93)
	2015–16	65.25% (61.20–69.29)	65.42% (56.28–74.57)	61.60% (50.53–72.67)	−3.82 (−18.28–10.64)	0.94 (0.75–1.18)
Arunachal Pradesh	2005–06	29.10% (20.87–37.34)	11.19% (−0.52–22.90)	49.81% (29.04–70.59)	38.63 (14.94–62.31)	4.45 (1.45–13.66)
	2015–16	38.30% (34.17–42.44)	25.18% (18.44–31.91)	42.80% (32.86–52.74)	17.62 (5.74–29.50)	1.70 (1.20–2.41)
Assam	2005–06	31.84% (23.66–40.03)	7.90% (−1.14–16.95)	60.44% (46.33–74.56)	52.54 (37.41–67.67)	7.65 (2.49–23.44)
	2015–16	48.48% (45.66–51.29)	35.80% (30.60–41.00)	64.88% (58.89–70.86)	29.08 (21.22–36.93)	1.81 (1.53–2.15)
Bihar	2005–06	32.83% (26.79–38.86)	13.99% (6.30–21.67)	64.97% (52.29–77.64)	50.98 (35.67–66.29)	4.64 (2.56–8.43)
	2015–16	62.54% (60.78–64.31)	53.44% (49.59–57.28)	71.20% (67.34–75.06)	17.76 (12.24–23.28)	1.33 (1.22–1.46)
Chhattisgarh	2005–06	48.72% (42.26–55.18)	29.85% (18.96–40.74)	88.14% (79.30–96.97)	58.29 (44.55–72.03)	2.95 (2.03–4.29)
	2015–16	76.90% (74.19–79.60)	61.34% (55.07–67.61)	89.46% (84.80–94.12)	28.12 (20.30–35.94)	1.46 (1.30–1.64)
Delhi	2005–06	63.19% (55.04–71.33)	32.49% (15.04–49.93)	78.66% (65.75–91.58)	46.18 (24.47–67.88)	2.42 (1.38–4.25)
	2015–16	69.13% (59.44–78.82)	55.04% (35.20–74.88)	69.80% (53.95–85.64)	14.76 (−11.12–40.64)	1.27 (0.82–1.96)
Goa	2005–06	78.57% (72.91–84.22)	62.19% (47.88–76.50)	96.75% (90.36–103.15)	34.57 (18.78–50.36)	1.56 (1.22–1.98)
	2015–16	88.44% (81.06–95.82)	78.33% (55.99–100.66)	100%	21.67 (−0.66–44.01)	1.28 (0.96–1.70)
Gujarat	2005–06	45.54% (38.10–52.98)	24.97% (10.96–38.98)	72.59% (59.31–85.88)	47.62 (28.06–67.18)	2.91 (1.60–5.27)
	2015–16	50.56% (47.11–54.00)	39.14% (33.24–45.03)	65.83% (57.26–74.40)	26.69 (16.36–37.02)	1.68 (1.38–2.05)
Haryana	2005–06	65.28% (55.50–75.07)	26.09% (11.16–41.02)	89.07% (78.07–100.07)	62.98 (45.60–80.36)	3.41 (1.93–6.04)
	2015–16	62.52% (59.01–66.03)	49.70% (42.41–56.99)	72.24% (64.40–80.09)	22.55 (11.46–33.63)	1.45 (1.20–1.76)
Himachal Pradesh	2005–06	74.18% (67.12–81.24)	48.43% (29.81–67.06)	84.20% (69.94–98.45)	35.76 (12.58–58.95)	1.74 (1.15–2.64)
	2015–16	69.82% (65.48–74.16)	68.69% (58.22–79.16)	77.78% (69.01–86.55)	9.09 (−4.64–22.81)	1.13 (0.94–1.37)
Jammu & Kashmir	2005–06	66.68% (60.23–73.14)	47.58% (31.34–63.83)	86.09% (76.41–95.78)	38.51 (18.83–58.19)	1.81 (1.25–2.62)
	2015–16	75.55% (72.69–78.41)	65.88% (60.34–71.41)	79.90% (72.99–86.81)	14.02 (5.20–22.85)	1.21 (1.08–1.37)
Jharkhand	2005–06	34.60% (26.48–42.72)	19.65% (9.73–29.58)	69.70% (55.68–83.73)	50.05 (33.68–66.43)	3.55 (2.10–6.00)
	2015–16	62.90% (60.44–65.36)	51.97% (47.11–56.84)	68.75% (63.09–74.41)	16.77 (9.33–24.22)	1.32 (1.17–1.50)
Karnataka	2005–06	55.24% (48.57–61.91)	20.72% (10.72–30.72)	72.62% (61.24–84.01)	51.90 (37.34–66.46)	3.50 (2.13–5.75)
	2015–16	63.14% (59.05–67.22)	61.24% (55.19–67.29)	62.52% (46.32–78.73)	1.28 (−16.01–18.56)	1.02 (0.77–1.35)
Kerala	2005–06	75.27% (68.04–82.51)	54.00% (35.03–72.97)	90.9% (80.84–100.95)	36.89 (15.19–58.60)	1.68 (1.16–2.44)
	2015–16	82.13% (78.07–86.19)	85.16% (77.21–93.11)	84.28% (75.46–93.10)	−0.88 (−12.95–11.18)	0.99 (0.86–1.14)
Madhya Pradesh	2005–06	40.32% (34.33–46.31)	24.05% (15.71–32.40)	84.25% (73.79–94.71)	60.2 (45.86–74.54)	3.50 (2.38–5.15)
	2015–16	53.85% (52.04–55.65)	36.77% (33.44–40.11)	69.35% (65.01–73.69)	32.58 (27.12–38.04)	1.89 (1.69–2.10)
Maharashtra	2005–06	59.03% (53.29–64.78)	45.96% (33.14–58.78)	72.57% (60.93–84.21)	26.61 (8.70–44.52)	1.58 (1.13–2.20)
	2015–16	56.62% (53.14–60.10)	51.39% (44.24–58.55)	60.40% (50.87–69.93)	9.01 (−3.90–21.91)	1.18 (0.94–1.48)
Manipur	2005–06	46.79% (39.64–53.94)	20.88% (9.19–32.58)	82.46% (73.42–91.50)	61.57 (47.16–75.99)	3.95 (2.24–6.95)
	2015–16	66.53% (63.09–69.96)	36.05% (29.56–42.53)	86.66% (82.11–91.21)	50.61 (42.73–58.50)	2.40 (1.99–2.90)
Meghalaya	2005–06	32.85% (23.93–41.78)	22.00% (5.77–38.24)	40.73% (24.15–57.30)	18.72 (−4.23–41.68)	1.85 (0.80–4.27)
	2015–16	61.81% (57.79–65.84)	50.92% (43.10–58.75)	76.75% (66.49–87.02)	25.83 (12.88–38.78)	1.51 (1.23–1.85)
Mizoram	2005–06	46.48% (36.35–56.61)	18.40% (−1.02–37.82)	73.13% (59.08–87.19)	54.73 (31.14–78.32)	3.97 (1.37–11.55)
	2015–16	50.87% (45.42–56.32)	38.91% (30.48–47.34)	64.05% (45.04–83.06)	25.14 (4.37–45.92)	1.65 (1.14–2.38)
Nagaland	2005–06	20.95% (15.94–25.97)	4.67% (0.10–9.24)	50.00% (37.00–63.00)	45.33 (31.55–59.10)	10.71 (3.89–29.48)
	2015–16	36.59% (32.54–40.64)	20.77% (14.78–26.76)	54.67% (43.80–65.53)	33.89 (21.40–46.38)	2.63 (1.85–3.74)
Odisha	2005–06	51.81% (44.92–58.70)	34.22% (19.31–49.13)	75.87% (66.60–85.14)	41.65 (24.22–59.08)	2.22 (1.41–3.48)
	2015–16	78.70% (76.40–80.99)	69.41% (64.43–74.40)	79.72% (73.51–85.93)	10.31 (2.34–18.27)	1.15 (1.03–1.28)
Punjab	2005–06	60.08% (52.69–67.47)	30.52% (16.58–44.47)	93.25% (85.67–100.84)	62.73 (45.34–80.12)	3.06 (1.89–4.95)
	2015–16	89.05% (86.62–91.47)	80.71% (74.24–87.17)	97.78% (94.89–100.66)	17.07 (10.03–24.11)	1.21 (1.11–1.32)
Rajasthan	2005–06	26.48% (20.86–32.10)	16.19% (6.06–26.31)	54.59% (41.68–67.49)	38.40 (22.43–54.37)	3.37 (1.75–6.50)
	2015–16	54.97% (52.82–57.12)	42.19% (37.99–46.38)	65.84% (60.68–71.00)	23.65 (17.06–30.25)	1.56 (1.38–1.77)
Sikkim	2005–06	69.64% (59.34–79.93)	54.94% (31.83–78.06)	85.12% (66.55–103.68)	30.17 (0.63–59.71)	1.55 (0.97–2.48)
	2015–16	83.01% (77.78–88.24)	82.30% (71.63–92.97)	79.42% (64.69–94.15)	−2.88 (− 21.06–15.29)	0.96 (0.77–1.21)
Tamil Nadu	2005–06	80.89% (75.24–86.54)	76.12% (61.12–91.11)	83.92% (71.77–96.06)	7.80 (−11.40–27.00)	1.10 (0.86–1.41)
	2015–16	70.07% (67.11–73.03)	68.24% (62.00–74.48)	75.46% (68.17–82.75)	7.22 (−2.41–16.84)	1.11 (0.97–1.26)
Telangana^c^	2005–06	–	–	–	–	–
	2015–16	67.56% (62.34–72.79)	64.11% (55.64–72.59)	71.90% (57.95–85.85)	7.78 (−8.35–23.92)	1.12 (0.89–1.41)
Tripura	2005–06	49.69% (37.78–61.60)	13.77% (2.06–25.49)	75.27% (56.05–94.48)	61.49 (36.92–86.07)	5.47 (2.14–13.99)
	2015–16	54.47% (47.26–61.67)	40.97% (25.86–56.08)	61.05% (45.37–76.73)	20.08 (−1.26–41.43)	1.49 (0.96–2.32)
Uttar Pradesh	2005–06	23.23% (20.11–26.35)	8.84% (5.24–12.45)	46.41% (37.79–55.02)	37.56 (28.38–46.74)	5.25 (3.38–8.15)
	2015–16	51.70% (50.29–53.12)	39.95% (37.00–42.89)	63.72% (60.55–66.90)	23.78 (19.46–28.10)	1.60 (1.46–1.74)
Uttarakhand	2005–06	60.04% (52.26–67.83)	28.12% (15.29–40.94)	81.82% (69.27–94.37)	53.70 (35.48–71.93)	2.91 (1.79–4.73)
	2015–16	57.89% (54.48–61.30)	46.67% (38.89–54.44)	70.94% (63.10–78.77)	24.27 (13.13–35.41)	1.52 (1.24–1.86)
West Bengal	2005–06	64.26% (57.32–71.20)	60.12% (48.67–71.58)	84.24% (72.65–95.82)	24.11 (8.20–40.02)	1.40 (1.11–1.76)
	2015–16	84.74% (82.22–87.27)	85.00% (80.35–89.64)	82.09% (74.98–89.21)	−2.90 (−11.45–5.65)	0.97 (0.87–1.07)
India	2005–06	43.65% (42.11, 45.19)	24.44% (21.76, 27.11)	70.97% (67.98, 73.95)	46.53 (42.53, 50.53)	2.90 (2.58, 3.26)
	2015–16	62.46% (61.78, 63.13)	53.42% (52.22, 54.63)	70.25% (68.34, 72.17)	16.83 (14.55, 19.12)	1.32 (1.27, 1.36)

^a^Calculated by subtracting the full immunization coverage in quintile 1 from coverage in quintile 5; ^b^Calculated by dividing the full immunization coverage in quintile 5 by coverage in quintile 1; ^c^Telangana separated from Andhra Pradesh in June 2014 and therefore immunization coverage for Telangana and Andhra Pradesh is reported together for 2005–06 but separately for 2015–16

**Fig. 2:**
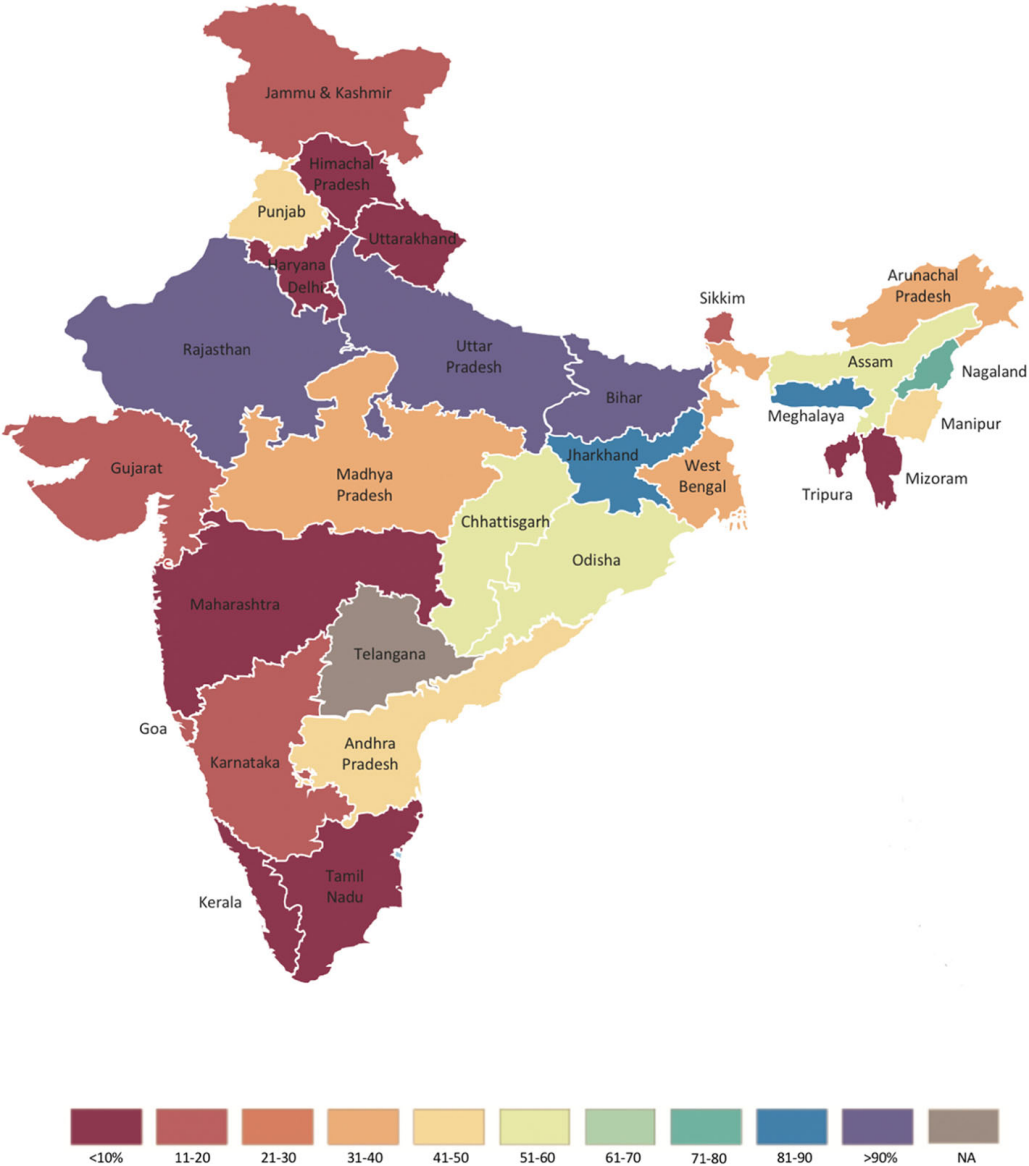
Change in full immunization coverage between 2005–06 and 2015–16 in India

The increase in national full immunization coverage is largely attributable to substantial increases in several populous states in the central and east regions of the country. Figure 2 describes state-level changes in full immunization coverage between 2005–06 and 2015–16. Full immunization coverage increased by more than 100% in two states: Uttar Pradesh in the North region (122.55% [95% CI: 90.85, 164.15%]) and Rajasthan in the Central region (107.59% [69.7, 173.83%]). Full immunization coverage in Bihar, a state in the East region, increased by 90.50% (95% CI: 56.41, 140.05%) during the same timeframe. Full immunization coverage decreased between 2005–06 and 2015–16 in five states; however, coverage was already relatively high in most of these states.

**Fig. 3:**
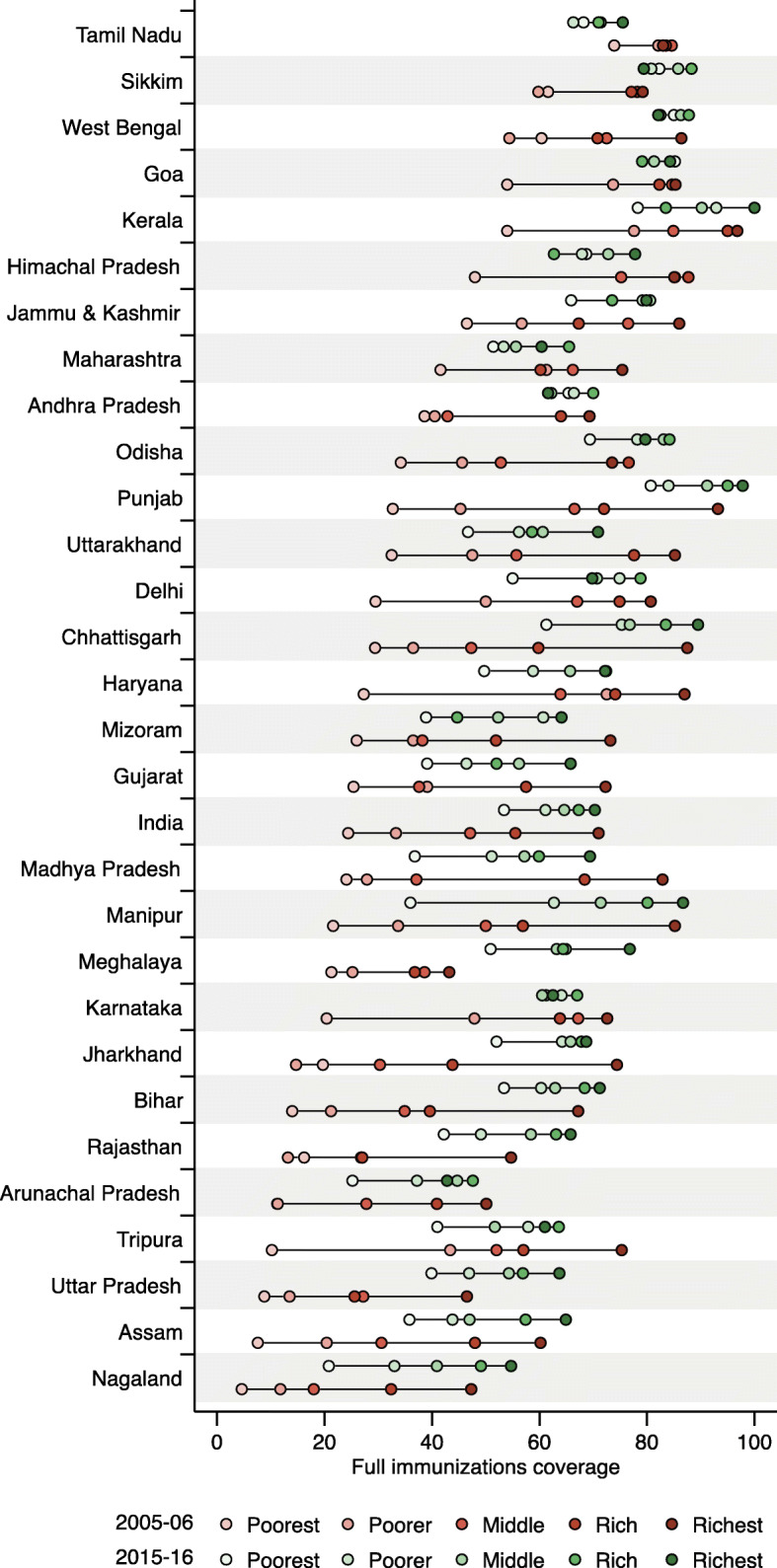
Full immunization coverage by wealth for 2005–06 and 2015–16 in India and by state

Overall, full immunization coverage in both 2005–06 and 2015–16 was generally lowest in children from poorer households and improved with increasing socioeconomic status suggesting pro-rich inequality (Table 2 and Fig. 3). At the national level, full immunization coverage was 24.44% (95% CI: 21.76, 27.11%) in the poorest wealth quintile compared with 70.97% (67.98, 73.95%) in the richest wealth quintile in 2005–06. Full immunization coverage increased to 53.42% (52.22, 54.63%) in the poorest quintile but remained similar in richest quintile 70.25% (68.34, 72.17%) in 2015–2016.

Table 3 describes the concentration indices for full immunization coverage at the national and state levels for 2005–06 and 2015–16. The national concentration index decreased substantially between the two survey periods from 0.36 (95% CI: 0.34, 0.38) to 0.13 (0.12, 0.14; *p*-value for difference = < 0.001). This indicates a statistically significant reduction in poor-rich inequalities for full immunization coverage nationally between 2005–06 and 2015–16.

**Table 3:** Full immunization concentration index estimates in 2005–06 and 2015–16 in all states

State	2005–06 concentration index (95% CI)	2015–16 concentration index (95% CI)	Absolute difference^*^
Andhra Pradesh	0.23 (0.12, 0.33)	−0.01 (−0.10, 0.08)	0.24***
Arunachal Pradesh	0.35 (0.19, 0.50)	0.17 (0.10, 0.24)	0.18*
Assam	0.39 (0.27, 0.51)	0.22 (0.17, 0.27)	0.17*
Bihar	0.37 (0.27, 0.47)	0.14 (0.11, 0.17)	0.24***
Chhattisgarh	0.43 (0.30, 0.55)	0.21 (0.16, 0.25)	0.22***
Delhi	0.38 (0.24, 0.52)	0.14 (0.03, 0.26)	0.24**
Goa	0.32 (0.20, 0.44)	0.08 (−0.07, 0.24)	0.24*
Gujarat	0.40 (0.28, 0.53)	0.18 (0.12, 0.24)	0.23***
Haryana	0.42 (0.29, 0.56)	0.19 (0.14, 0.25)	0.23**
Himachal Pradesh	0.20 (0.06, 0.34)	0.04 (−0.05, 0.12)	0.17*
Jammu & Kashmir	0.28 (0.15, 0.42)	0.08 (0.04, 0.13)	0.20**
Jharkhand	0.40 (0.28, 0.52)	0.13 (0.08, 0.17)	0.27***
Karnataka	0.35 (0.24, 0.46)	0.02 (−0.04, 0.07)	0.33***
Kerala	0.26 (0.13, 0.40)	−0.01 (−0.09, 0.07)	0.27***
Madhya Pradesh	0.43 (0.34, 0.52)	0.23 (0.20, 0.27)	0.19***
Maharashtra	0.20 (0.12, 0.29)	0.10 (0.05, 0.15)	0.11*
Manipur	0.45 (0.34, 0.57)	0.39 (0.34, 0.45)	0.06
Meghalaya	0.17 (0.03, 0.32)	0.16 (0.09, 0.24)	0.01
Mizoram	0.41 (0.24, 0.58)	0.10 (0.03, 0.17)	0.31***
Nagaland	0.33 (0.24, 0.41)	0.28 (0.21, 0.34)	0.05
Odisha	0.37 (0.25, 0.50)	0.09 (0.05, 0.13)	0.28***
Punjab	0.47 (0.34, 0.61)	0.15 (0.10, 0.19)	0.33***
Rajasthan	0.27 (0.17, 0.38)	0.20 (0.16, 0.24)	0.07
Sikkim	0.19 (0.01, 0.37)	0.01 (−0.11, 0.13)	0.18*
Tamil Nadu	0.05 (−0.05, 0.15)	0.06 (0.01, 0.11)	−0.01
Tripura	0.50 (0.31, 0.69)	0.15 (0.01, 0.29)	0.35**
Uttar Pradesh	0.28 (0.22, 0.33)	0.18 (0.16, 0.21)	0.10**
Uttarakhand	0.45 (0.31, 0.59)	0.15 (0.08, 0.21)	0.30***
West Bengal	0.19 (0.09, 0.29)	0.01 (−0.05, 0.05)	0.19***
India	0.36 (0.34, 0.38)	0.13 (0.12, 0.14)	0.23***

^*^Calculated by subtracting the concentration index in 2005–06 from the concentration index in 2015–2015; *** = *p*-value < 0.001; ** = *p*-value < 0.01; * = *p*-value < 0.05

Poor-rich inequalities in most states decreased between 2005–06 and 2015–16. The greatest reduction in inequality was in the state of Karnataka in the South where the concentration index decreased from 0.35 (95% CI: 0.24, 0.46) in 2005–06 to 0.02 (− 0.04, 0.07) in 2015–16 (*p*-value for difference = < 0.001). As measured by the concentration index, poor-rich inequalities increased in only one state (i.e., Tamil Nadu in the South), but the increase was not statistically significant. In 2015–16, states with high full immunization poor-rich inequalities were Manipur in the Northeast region (0.39 [0.34, 0.45]), Nagaland also in the Northeast (0.28 [0.21, 0.34]), Madhya Pradesh in the Central region (0.23 [0.20, 0.27]), and Assam in the Northeast region (0.22 [0.17, 0.27]). Full immunization coverage was pro-poor in Andhra Pradesh (− 0.01, [− 0.10, 0.08]) and Kerala (− 0.01 [(− 0.09, 0.07)]) in 2015–16, both states in the South region.

**Fig. 4:**
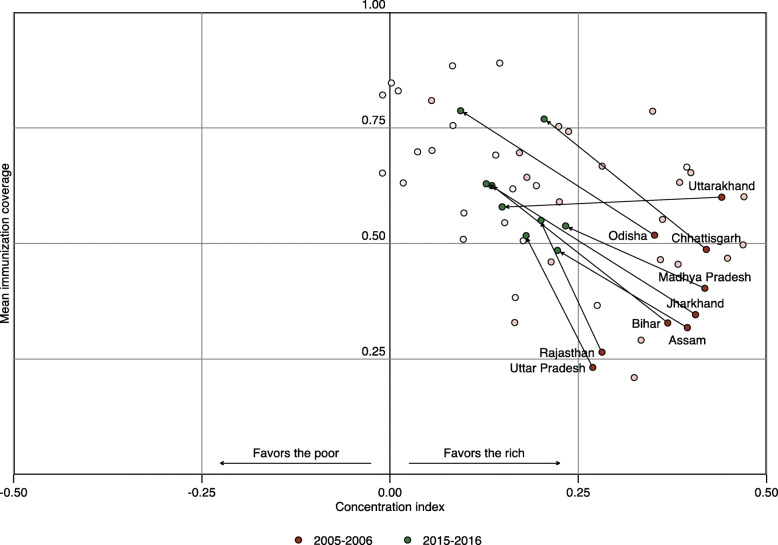
Change in concentration indices and mean state coverage between 2005–06 and 2015–16 by state, with details provided for high-focus states. Nine high-focus states are labelled with arrows connecting 2005–06 estimates (maroon) with 2015–16 estimates (forest green)

We assessed changes in full immunization inequality for nine states with high infant mortality that together have been designated as high-­focus states by the Government of India (Fig. 4). Full vaccine coverage increased for each of the high-­focus states between 2005–06 and 2015–16, except for Uttarakhand in the North region, for which full immunization coverage decreased by 3.58% (95% CI: −19.68, 17.30%). The concentration index for all high-focus states decreased significantly toward 0, indicating an increase toward favoring the poor, during this timeframe, except in Rajasthan where the decrease in concentration index was not statistically significant. Uttarakhand in the North region represented the greatest reduction in poor-rich inequalities among high-focus states, with the concentration index decreasing from 0.45 (0.31, 0.59) in 2005–06 to 0.149 in 2015–16 (*p*-value < 0.001). Full immunization coverage continues to favor the rich in all high-focus states.

Several predictors individually contribute to 5% or more of the overall concentration index (Table 4). In 2005–06, the primary contributors to wealth-related full immunization inequality were antenatal care (13.08%), maternal education (9.03%), and socioeconomic status (6.78%). These same factors were associated with wealth-related inequalities in full immunization coverage in 2015–16 contributing to 38.46, 7.30, and 10.62% of wealth-related inequalities in full immunization coverage, respectively. In 2015–16, difficulty in reaching a health facility also contributed to 8.45% of the overall concentration index. Several factors in 2005–06 and 2015–16 had a negative contribution to the overall concentration index, indicating they contributed to a reduction in the wealth-related inequality for full immunization coverage. These factors include region and geographic setting (i.e., urban or rural location).

**Table 4:** Elasticities, concentration indices, and contributions of determinants to wealth-related inequality for full immunization status in India in 2005–06 and 2015–16

Determinants of full immunization	2005–06				2015–16			
	Elasticity	Concentration Index	Contribution	Percent contribution	Elasticity	Concentration Index	Contribution	Percent Contribution
**Gender: Female**	−0.05*	−0.04	0.00	**0.56%**	−0.02	0.01	0.00	**−0.11%**
**Birth order (ref: First)**	–	–	–	**2.83%**	–	–	–	**1.93%**
Second	0.11	−0.08	− 0.01	−0.24%	0.08*	−0.01	0.00	−0.62**%**
Third	−0.01	−0.02	0.00	0.19%	−0.08***	−0.01	0.00	0.90**%**
Fourth	−0.07	−0.01	0.00	0.10%	−0.08***	−0.01	0.00	0.52**%**
Fifth or greater	−0.21**	−0.05	0.01	2.78%	−0.11***	−0.01	0.00	1.13**%**
**Religion (ref: Hindu)**	–	–	–	**−0.08%**	–	–	–	**−0.15%**
Muslim	0.01***	−0.04	0.00	−0.08%	0.02***	−0.02	0.00	−0.29**%**
Other	0.04	0.00	0.00	> 0.01%	0.04***	0.00	0.00	0.14**%**
**Caste (ref: general)**	–	–	–	**2.67%**	–	–	–	**0.01%**
SC/ST	−0.29	−0.03	0.01	2.70%	−0.27	0.00	0.00	−0.31**%**
OBC	0.00	−0.05	0.00	−0.02%	0.06	0.01	0.00	0.32**%**
**Wealth quintile (ref: Poorest)**	–	–	–	**6.78%**	–	–	–	**10.62%**
Poorer	−0.25	0.03	−0.01	−1.96%	−0.25***	0.02	0.00	−3.35**%**
Middle	0.11***	0.05	0.01	1.38%	0.10***	0.02	0.00	1.28**%**
Richer	0.34**	0.03	0.01	2.90%	0.38***	0.02	0.01	6.12**%**
Richest	0.53***	0.03	0.02	4.45%	0.51***	0.02	0.01	6.57**%**
**Maternal education (ref: no education)**	–	–	–	**9.03%**	–	–	–	**7.30%**
Incomplete primary	−0.03***	0.02	0.00	−0.13%	−0.07***	0.01	0.00	−0.42**%**
Complete primary	0.00***	0.02	0.00	0.01%	−0.04***	0.01	0.00	−0.21**%**
Incomplete secondary	0.35***	0.08	0.03	8.20%	0.14***	0.04	0.01	4.50**%**
Complete secondary	0.10***	0.01	0.01	0.25%	0.13***	0.01	0.00	1.02**%**
Higher	0.17***	0.02	0.00	0.70%	0.27***	0.01	0.00	2.41**%**
**Antenatal care (ref: no visits)**	–	–	–	**13.08%**	–	–	–	**38.46%**
Insufficient (i.e., < four visits)	−0.18***	0.24	−0.04	−12.01%	−0.16***	0.08	−0.01	−9.99**%**
Sufficient (i.e., ≥ four visits)	0.51***	0.18	0.09	25.09%	0.38***	0.16	0.06	48.45**%**
**Geographic setting: Rural**	−0.49	0.03	−0.01	**−3.97%**	− 0.50***	0.05	− 0.02	**−18.41%**
**Difficulty reaching a health facility (ref: no problem)**	–	–	–	**4.95%**	–	–	–	**8.45%**
Not a big problem	−0.07	−0.01	0.00	0.12%	−0.04**	−0.01	0.00	0.44**%**
Big problem	−0.35*	−0.05	0.02	4.83%	−0.29***	−0.04	0.01	8.01**%**
**Region (ref: Central)**	–	–	–	**−5.15%**	–	–	–	−**14.32%**
East	−0.22***	0.14	−0.03	−8.46%	−0.32***	0.06	−0.02	−14.53**%**
North	0.11***	0.02	0.00	0.66%	0.15***	0.01	0.00	1.11**%**
Northeast	−0.01	0.00	0.00	> 0.01%	−0.03***	−0.01	0.00	0.12**%**
South	0.15***	0.04	0.01	1.54%	0.20	0.00	0.00	0.61**%**
West	0.10***	0.03	0.00	1.11%	0.11***	−0.02	0.00	−1.64**%**

*** = *p*-value < 0.001; ** = *p*-value < 0.01; * = *p*-value < 0.05

## Discussion

We measured changes in national- and state-level immunization coverage inequalities in children 12–23 months over 10 years from 2005–06 (NFHS-3) to 2015–2016 (NFHS-4) and the contributors to inequalities in India. Our results indicate that full immunization coverage increased substantially during this timeframe and the between state differences in full immunization coverage also decreased. We found that equality increased in India nationally and within almost every state during the decade. Several factors contributed to wealth-related inequalities in full immunization coverage, including socioeconomic status, maternal education, and antenatal care in both 2005–06 and 2015–16.

Globally, previous studies have established a strong link between poor health outcomes and low socioeconomic status, poor education, and limited access to health care [26]. In India, disparities in under-five mortality by wealth are among the highest in the world, with the under-five mortality differing by more than 30 deaths per 1000 live births between the highest and poorest wealth quintiles in 2016 [27]. Children living in disadvantaged households generally have higher rates of important risk factors for infectious diseases, including malnutrition, inadequate water and sanitation, and indoor air pollution [28]. Further, these children are less likely to have access to preventative services, including immunization, and treatment should they become sick—only 73.2% of children in 2015–16 with acute respiratory infection symptoms were taken to a health facility [4].

Our findings are consistent with other studies on full immunization inequalities and studies that assessed contributing factors to wealth-related immunization inequalities at the national level in India. One recent study found that pro-rich inequality decreased between 2005–06 and 2015–16 at the national level [29]. However, state-level estimates of inequality were not assessed in this analysis. In addition, other studies from India have found associations between full immunization coverage and several demographic, socioeconomic, and access to antenatal and postnatal care services at the national and in states [8, 30–32].

Some previously published studies have decomposed wealth-related immunization inequalities using data from NFHS-3, NFHS-4, and other previous datasets [33–37]. One such study used the concentration index and decomposition to the contributors to full immunization inequality at the national-level using the NFHS-4 data, finding a positive associations with socioeconomic states, literacy, institutional delivery, place of residence, and geographic location (i.e., urban versus rural) [37]. A meta-analysis of studies found that children with mothers who received secondary education or higher, compared to those with no education, had 2.3 times the odds of being fully immunized [38]. The association between maternal education and likelihood of being partially or unvaccinated has also been observed in India, with one study highlighting the role of health knowledge as a mediator between maternal education and immunization status [39, 40].

The associations identified in this study suggest the need for complementary strategies to increase utilization of care across the continuum of care from reproductive health services through to childhood and adolescence. The multiple-strategy community interventions that comprise the NHM have been demonstrated to be effective at reducing inequalities in several health outcomes, including immunization [41–43]. The PRECEDE/PROCEED framework has been used for intervention planning to address a range of health challenges in low- and middle-income settings, including immunization [44]. Specifically, the underlying approach entails identifying a desired result (e.g., immunization equality), determining the causes, and designing an intervention to achieve the desired result [15]. Understanding the root causes of immunization inequalities will help policy makers and program managers develop strategies to increase health service coverage and eliminate barriers for underserved populations.

The timing of most recent NFHS survey data collection prevented us from investigating the full impact of Mission Indradhanush on immunization coverage. Launched in 2014, Mission Indradhanush aims to increase full immunization coverage for all children to 90% [45]. The program was designed to target underserved, vulnerable, and hard-to-reach populations to close coverage gaps and reduce inequalities at local levels [46]. Mission Indradhanush has increased full immunization coverage nationally by 6.7%**—**7.9% in rural and 3.1% urban areas**—**according to the Ministry of Health and Family Welfare. Additional phases of the program are underway, by the name Intensified Mission Indradhanush 2.0, for 272 districts and 27 states in 2020 [47]. Rigorous program evaluations, which have not been conducted according to our knowledge, could provide valuable data on the effectiveness of this effort and potential adaptions for the future.

Strengths of this study included estimation of changes in full immunization coverage and inequalities over a 10-year period between two large nationally representative surveys (i.e., NFHS done in 2005–06 and 2015–16). Given the disparities in coverage by state, we assessed immunization coverage inequalities within each state by calculating concentration indices. This study also has limitations. Our outcome in this analysis, full immunization coverage, was in some cases reliant upon maternal recall, which has been demonstrated to have variable sensitivity and specificity for assessing immunization coverage in several countries, including India [48, 49]. In NFHS-3 and NFHS-4, observed immunization cards were the source of immunization status for only 38 and 63% of respondents, respectively. In addition to the introduction of measurement bias, the substantial increase in the availability of immunization between survey rounds could undermine the ability to make reliable comparisons over time. Some potentially important predictors of full immunization coverage were not measured in the surveys, including indicators of health system strength, trust in health services, or vaccine confidence. Lastly, several important vaccinations, including the pneumococcal conjugate vaccine (PCV), rotavirus vaccine (RVV), and rubella vaccine have only been recently introduced in India and data were not available in the NFHS surveys in this analysis. Future studies could evaluate coverage and inequalities for these critical vaccinations.

## Conclusions

Immunization coverage and wealth-related equality have improved nationally and in most states over the last decade in India. As publicly funded initiatives aim to deliver services on the basis of need rather than ability to pay, the government’s commitment to additional rounds of Mission Indradhanush will help improve coverage among the hardest to reach communities in India. This will help to further reduce wealth-related immunization inequalities. Yet substantial inequalities—across geography, socioeconomic factors, and access to care—still exist. Those who are still not fully immunized represent those with higher risk of infectious diseases independent of vaccination status. Our results suggest that targeted, context-specific interventions at the state- and district-levels will be needed to increase immunization coverage to sufficiently high levels required to prevent transmission of vaccine-preventable diseases and reduce wealth-related inequalities. Future research should aim to evaluate health policies and the effectiveness of programs tailored to specific populations based on immunization coverage, demand, and confidence in this essential health intervention. In addition, this study indicates that some social determinants of health (i.e., wealth and education) continue to be associated with immunization inequalities in India. The impact of broader government policies on full immunization coverage inequalities should also be quantified and contextualized in further research.

## Data Availability

Data are available publicly from the DHS Program website: https://dhsprogram.com. Code used for analyses is available upon request. Figure.

## Abbreviations

DTP: Diphtheria-tetanus-pertussis vaccine
BCG: Bacillus Calmette–Guérin vaccine
NFHS: National Family Health Survey
NRHM: National Rural Health Mission
NHM: National Health Mission
NUHM: National Urban Health Mission
EPI: Expanded Programme on Immunization
UIP: Universal Immunization Programme
fIPV: Fractional dose inactivated polio vaccine
MR: Measles-rubella vaccine
OPV: Oral polio vaccine
Hib: *Haemophilus influenzae* type b
RVV: Rotavirus vaccine
TT: Tetanus toxoid
PCV: Pneumococcal conjugate vaccine
JE: Japanese encephalitis
MOHFW: Ministry of Health and Family Welfare
IIPS: International Institute for Population Science
SC/ST: Scheduled caste and scheduled tribe
GLM: Generalized linear model
CI: Confidence interval
